# Ascending Aorta Nose-Cone Loop Technique as Bail Out for Precise Branched Endovascular Aortic Arch Endograft Delivery Without Valve Re-Crossing

**DOI:** 10.1177/15266028231201532

**Published:** 2023-10-12

**Authors:** Alessandro Grandi, Tilo Kölbel, Fiona Rohlffs, Daour Yousef al Sarhan, Giuseppe Panuccio

**Affiliations:** 1 German Aortic Center Hamburg, University Heart Center, University Medical Center Hamburg-Eppendorf, Hamburg, Germany

**Keywords:** aortic arch, BEVAR, guidewire, brachio-femoral, steerable sheath, aortic dissection, bailout

## Abstract

**Purpose:**

To describe a right carotid-femoral through-and-through (T&T) guidewire technique during branched thoracic endovascular aortic arch repair (B-TEVAR) to facilitate endograft delivery in a very tortuous aortic anatomy for a type Ia endoleak (EL) of a previous aortic endograft implantation.

**Technique:**

AT&T guidewire was established between the right common carotid artery and the right common femoral artery to facilitate a difficult endograft delivery. Once in the aortic arch, a loop in the ascending aorta was formed to allow the endograft to reach the desired position without losing tension on the guidewire. This maneuver allowed the T&T guidewire to be kept in place until the desired position was reached. The nose-tip of the endograft was curved over the looped guidewire pointing toward the innominate artery without crossing the valve. After endograft deployment, the T&T guidewire was released, and the branches were bridged in a standard fashion. Completion angiography documented correct deployment of the endograft and no sign of type I/III EL. The 1-month computed tomography angiography confirmed the correct deployment.

**Conclusion:**

Carotid-femoral T&T guidewire to facilitate endograft delivery in difficult anatomies can be feasible even in B-TEVAR. Possible bailout maneuvers are available if the aortic valve needs to be crossed after endograft delivery.

**Clinical Impact:**

Endovascular arch repair gains popularity as a valuable alternative, especially in patients considered unfit for open repair. A through-and-through (T&T) guidewire for endovascular arch repair with a landing zone in zone 0 according to Ishimaru is usually performed through the externalization of the femoral guidewire through a transapical access, but this may not always be feasible in frail patients. A right carotid-femoral though-and-through guidewire with a loop formation in the ascending aorta is proposed to achieve the support of a T&T wire to pass tortuous aortoiliac anatomies and access the ascending aorta without the need for aortic valve crossing.

## Introduction

Branched thoracic endovascular aortic arch repair (B-TEVAR) is rapidly gaining traction, especially in patients with previous ascending replacement who are unfit for redo open procedures.^1^ When planning a procedure, great attention is required to aortic tortuosity, especially in patients with previously implanted aortic endografts as it could hamper endograft advancement. Different techniques have been proposed to alleviate this problem, such as the use of stiff wires to reduce the angulation, or a through-and-through (T&T) guidewire usually established to create a stiff rail between a brachial and a femoral access.^2^ This maneuver allows delivering a device even through severely kinked anatomy without risking to lose the wire position. The standard T&T guidewire for B-TEVAR is usually obtained with transapical access through which the femoral guidewire is externalized.^3^ Although commonly used, this approach is not always risk-free and feasible due to the patients’ clinical conditions.

We present a new bail out technique to establish a T&T guidewire with externalization of the femoral guidewire at the level of the right common carotid artery (RCCA) during B-TEVAR avoiding the need of a transapical approach.

## Procedure Details

**Figure 1.**
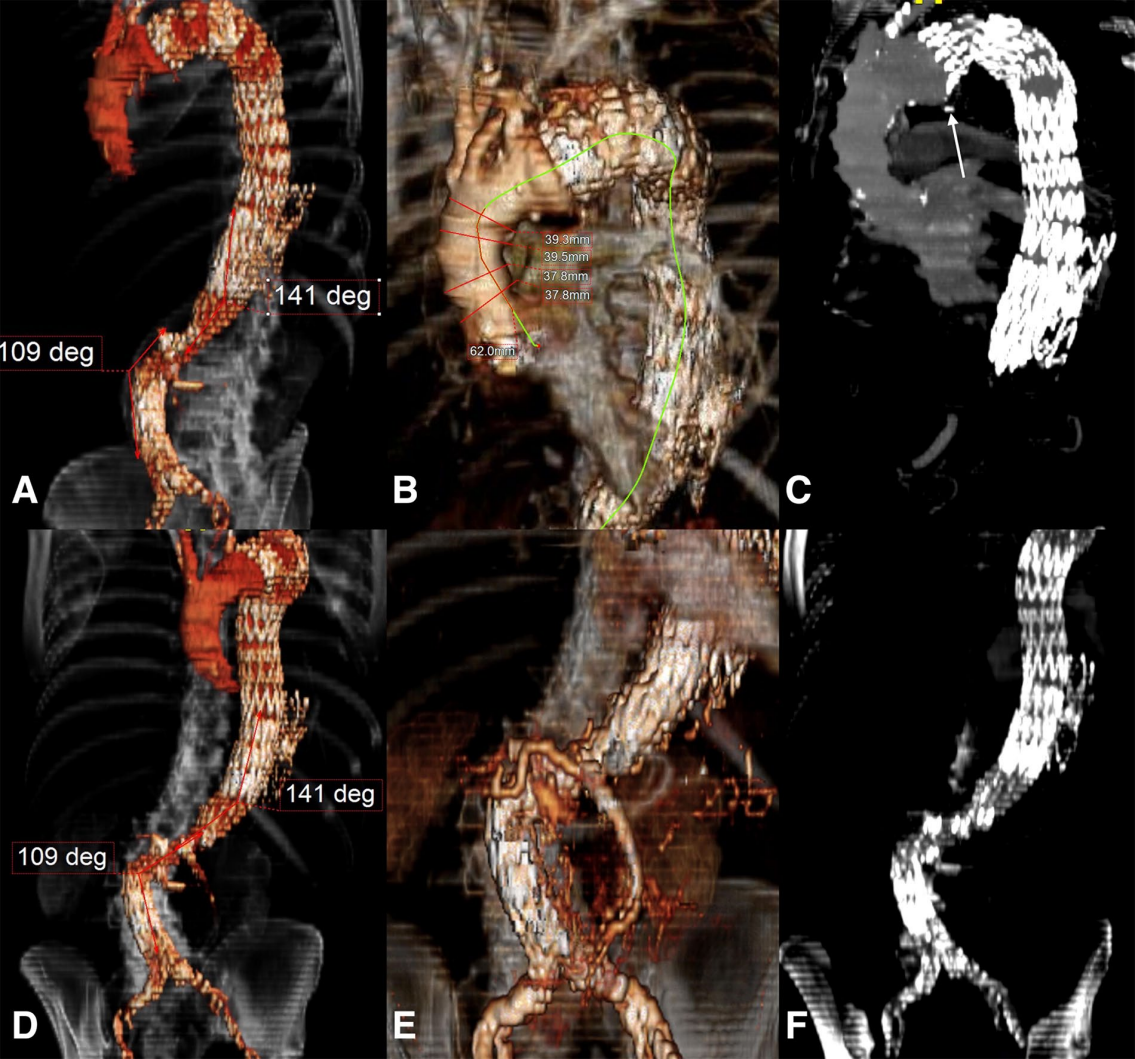
Preoperative computed tomography angiography showing (A and B) 3D and (C) multiplanar reconstruction of the migration of the previously implanted endograft, measurements (diameters and length) of the proximal sealing zone in the ascending aorta and angles of the infra-renal and supra-renal aorta are noted and the white arrow shows the retroflexion of the sealing stent of the previously implanted endograft, (D and E) 3D and (D) multiplanar reconstruction of the severe angulation (>90°) at the level of the infrarenal aorta.

A 65-year-old man with a history of hypertension suffered a type B aortic dissection in 2007. Due to false lumen aneurysmal degeneration, he was treated with a zone 2 TEVAR with a left carotid to subclavian bypass and left subclavian artery plug embolization, a Candy-Plug in the false lumen and a four-fenestration endovascular repair (FEVAR).^4^ During routine follow-up, the computed tomography angiogram (CTA) showed a degeneration of the proximal landing zone combined with distal migration of the previously implanted TEVAR resulting in a type Ia endoleak (EL) and a moderate kinking by retroflexion of the sealing stent. The same CTA showed a severe angulation of the level of the infrarenal aorta (>90°) (Figure 1). For this reason, a B-TEVAR was planned with a custom made 2-branched (both antegrade) arch endograft. A standard 60 mm long nose-cone was planned for the delivery system, which was 24F inner diameter (9.1 mm outer diameter).

Initially, a standard delivery technique was attempted and an extended double curved Lunderquist wire (Cook Medical, Bloomingdale, Indiana) was placed in the left ventricle.^5^ Despite multiple attempts and manipulation of the graft, delivery in the aortic arch was not achievable, followed by the loss of the wire position in the left ventricle.

### Bail Out Technique

**Figure 2.**
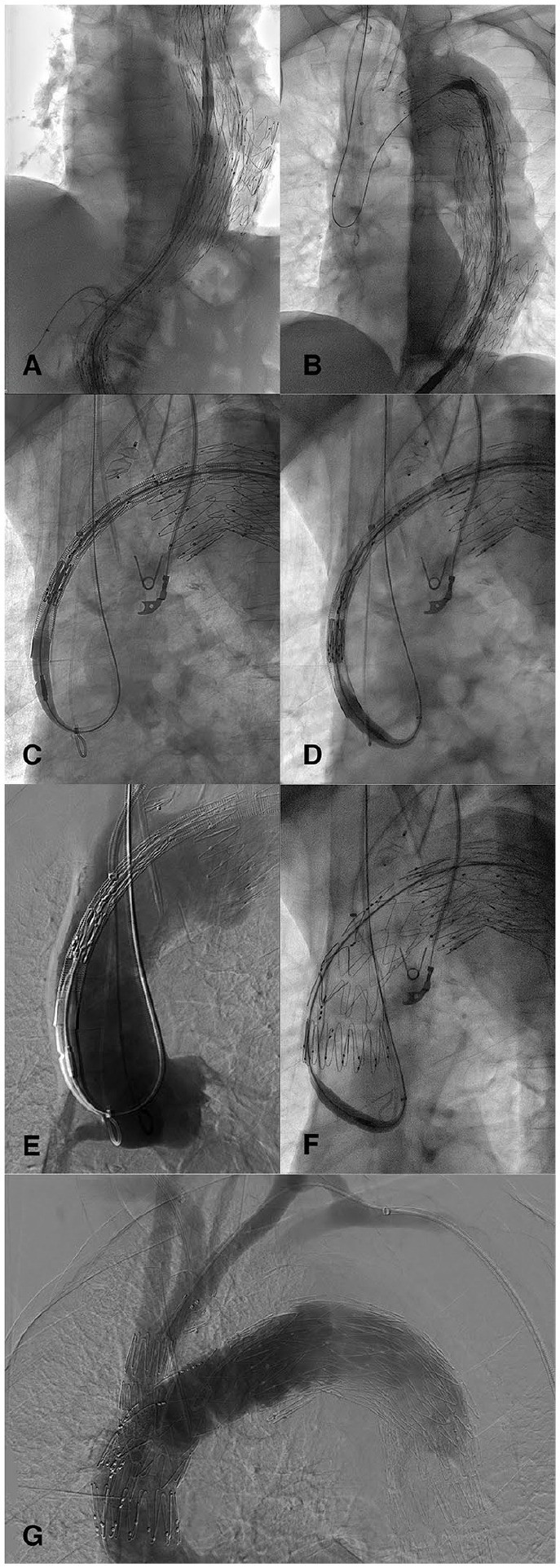
Intraoperative angiography images showing (A) an introducer sheath positioned inside the right renal artery stent to avoid damaging it during endograft delivery, (B) the through-and-through guidewire loop formation in the ascending aorta, (C) the endograft advancing in the ascending aorta over the looped through-and-through guidewire with the pigtail catheter positioned as an orientation marker, (D) the bending of the nose-tip of the endograft over the looped through-and-through guidewire pointing toward the innominate artery, with the nose-tip protected by an introducer sheath advanced from the innominate artery to avoid aortic valve damage, (E) the aortography to check the graft position before deployment, (F) correct deployment of the endograft at the intended position, and (G) completion angiography showing patency of target vessels and absence of type I/III endoleak.

Beside the standard vascular accesses already described in previous publication,^6^ a 7F×55 cm sheath (Cook Medical) was introduced in the RCCA after surgical exposure of the vessel. From this access, it was possible to snare the dislocated Lunderquist wire at the level of the ascending aorta establishing a T&T (7F Ensnare, Merit Medical System, South Jordan, UT). After establishing the T&T system, the 7F sheath was then advanced in the descending aorta in order to protect the aortic arch during the push and pull movement to advance the endograft in the desired position. At the same time, the right renal artery fenestration was catheterized from the left groin and a second 7F sheath was placed to prevent a possible stent deformation while overcoming the aortic kinking at the level of the renovisceral segment (Figure 2A). In a second step, while approaching the arch with the endograft, the 7F sheath from the RCCA was again placed in the ascending aorta, and the Lunderquist wire was looped over the aortic valve. This allowed the graft to advance through the aortic arch (Figure 2B). In the last step, pulling the T&T wire from the RCCA permitted the bending of the nose-cone in the direction of the innominate artery (IA). Thanks to this maneuver, it was possible to reach the desired proximal landing zone with the endograft (Figure 2C and D, Supplemental Video 1). Of note, a 5F pigtail catheter was kept in place at the level of the aortic valve as an orientation marker and to perform further angiographies (Figure 2E).

**Figure 3.**
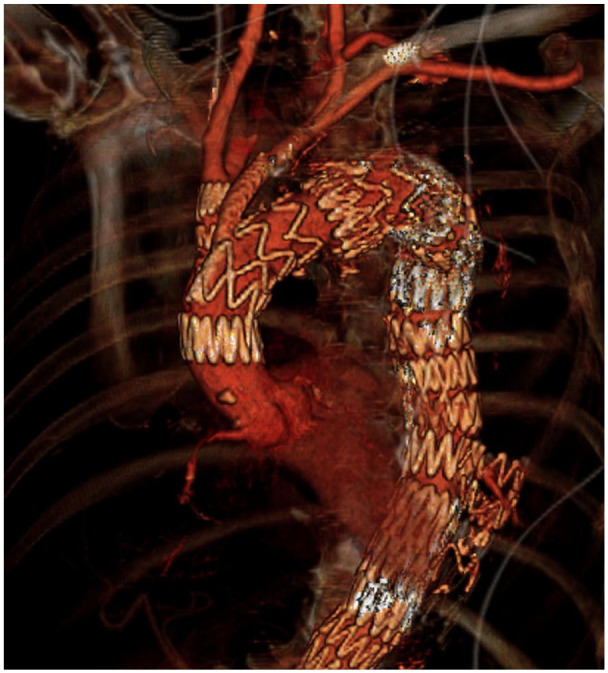
Three-dimensional reconstruction of the 1-month computed tomography angiography showing correct deployment of the endograft, patency of the branches and no signs of type I/III endoleak.

After reaching the intended delivery position, the endograft was deployed using the Munich Valsalva Implantation Technique (MuVIT)^7^ (Figure 2F) and the branches were bridged in a standard fashion,^5^ without any complications (Figure 2G). A 1-month follow-up CTA showed a fully patent graft with complete false lumen thrombosis without type I/III EL (Figure 3).

## Discussion

Aortic aneurysms are frequently associated with multiple tortuous segments of the aorta,^8^ which may not always be corrected with the use of stiff guidewires alone.

For standard TEVAR in the thoracic aorta or distal aortic arch, a brachio-femoral T&T guidewire represents an effective tool to advance endografts through tortuous anatomy by pulling the externalized guidewire and straightening the vessels.^9^ For aortic arch TEVAR with landing in zone 0, this is not possible as the nosecone will have to pass beyond the IA. A transapical or transseptal T&T wire may be used, but this remains an invasive adjunct requiring surgical apical exposure or an additional intracardiac puncture.^3,9^

In cases with severe tortuous anatomy, planning of the vascular access is of the utmost importance. Selecting the right device and introducer sheath design can mean the difference in reaching the intended deployment position or not. The length of the delivery system’s nose-cone is important to deal with the tortuosity of the iliac vessels but may represent a limitation at the level of the aortic arch and require the crossing of the aortic valve to deliver the endograft in zone 1 or 0. Most of the commercially available arch devices require crossing of the valve with the guidewire and the nose-cone. One alternative technique to the standard nose-cone’s length was a shorter proximal bullet-nose configuration as published by Spear et al,^10^ stating that the presence of a mechanical aortic valve was not a contraindication for the procedure anymore. Recently, a shorter version (35 mm) was introduced to allow zone 0 landing in patients with mechanical aortic valves,^11^ but this design may reduce the trackability of the system in very tortuous anatomy, which is why it was not chosen in the presented case.

Today, the commercially available Najuta stent graft (Kawasumi Inc.; Tokyo, Japan) does not require aortic valve crossing and the suggested delivery and deployment sequence is similar to the one reported in this case. A T&T is established and looped over the aortic valve, but due to the nose-cone being shorter, the need to bend, it is minimal.

The technique of bending the nosecone using a T&T wire loop combines the best of both the brachio-femoral T&T and the transapical approach. The creation of the loop in the ascending aorta at the level of the aortic valve, while protecting it with a catheter/sheath during the nose-cone bending, allows to achieve a similar active conformation of the endograft as the transapical approach. There is also an intrinsic lower risk of damaging the origin of the IA as the delivery system is never advanced into the IA. This technique adds to the armamentarium of approaches that may be used in patients with mechanical valves who need a B-TEVAR.

A further possible alternative in case of inability to bend the nose-tip over the looped stiff guidewire in the direction of the IA, would be the “snare-ride” technique.^12^ By snaring the looped stiff guidewire with a coaxial snare system (IndySnare, Cook Medical) from the IA and pushing the system across the aortic valve, the nose-cone can be advanced in a standard position for deployment, avoiding any possible drawbacks from not having crossed the valve sooner or having lost the wire position during delivery.

An important aspect for device trackability is the hydrofiling coating of the delivery system to allow a smooth passage of the endograft. The present technique does not require the extraction of the graft in case of wire position loss, therefore reducing the risk to lose the hydrophilic coating. This, associated with a carful maneuvering, would not increase the risk of access vessels injuries compared to a standard endograft delivery. Furthermore, every possible periprocedural step was taken to minimize iliac manipulation and at the end of the procedure, during the sheath removal, careful attention was paid to checking for iliac injuries by checking patient pressure and performing and angiographic check of the iliac arteries at the time of sheath removal.

The main limitation of the described approach remains the number of manipulations required at the level of the arch with an associated stroke risk (10%),^13^ as well as the trauma at the level of the ascending aorta resulting in a risk of aortic dissection when performed in a native aorta. Furthermore, the presented technique may not be generalized as different arch morphologies may hinder its use. Careful attention should be paid to the arch type and the characteristics of the supra-aortic vessels.

## Conclusion

The nose-cone loop technique with T&T guidewire reported has facilitated endograft delivery through challenging aortic anatomy and avoided the need to re-cross the aortic valve.
